# Surgical Treatment of Dural Arteriovenous Fistula: A Case Report and Literature Review

**DOI:** 10.7759/cureus.18995

**Published:** 2021-10-23

**Authors:** Denis Babici, Phillip Johansen, Brian Snelling

**Affiliations:** 1 Neurology, Charles E. Schmidt College of Medicine, Florida Atlantic University, Boca Raton, USA; 2 Neurosurgery, Boca Raton Regional Hospital, Boca Raton, USA

**Keywords:** endovascular intervention, catheter angiogram, 3d model in neurosurgery, brain vascular disease, dural arteriovenous fistulas

## Abstract

Dural arteriovenous fistulas (dAVF) are rare, acquired intracranial arteriovenous malformations consisting of a pathological shunt located within the intracranial dura matter. The etiology of dAVFs remains unclear, but current thought suggests that these lesions are associated with thrombosis of the dural sinuses and other intracranial veins. dAVF’s with severe symptomatology or high-risk angioarchitecture should be treated without delay, and endovascular repair is generally accepted as the first-line treatment. Both transarterial and transvenous approaches can be used to cure dAVFs. Surgery and stereotactic radiosurgery may also be used when endovascular approaches are unsuccessful or not feasible. Some studies, however, have shown that surgery for dAVFs in the anterior cranial fossa is preferred over the endovascular approach. Due to the proximity of some dAVFs to the orbit, endovascular embolization of the dAVF carries a higher risk of complications, primarily due to the formation of dangerous extracranial or intracranial anastomoses. We present the case of a 64-year-old male with an incidentally discovered Borden type III dAVF arising from the anterior branches of the middle meningeal artery and draining into the middle cerebral vein. Due to the location of his dAVF, craniotomy was selected for ligation of the fistula. The procedure went without complication. A catheter angiogram of the brain one month after surgery showed an absence of flow through the arteriovenous fistula and a middle meningeal artery that had returned to its normal caliber.

## Introduction

Dural arteriovenous fistulas (dAVFs) are intracranial, dural-based shunts that account for 10% - 15% of all intracranial arteriovenous malformations [1-2]. These fistulas generally derive from meningeal arteries and drain either through the dural venous sinuses or cortical veins [1]. dAVFs are most frequently seen in adults over the age of 60 [2]. Of note, dAVFs in males are more likely to present with hemorrhage and display aggressive neurological symptoms despite intracranial dAVFs being more common in females [3]. dAVFs may also occur in the pediatric population where they typically present with complex lesions that are often supplied by bilateral arterial feeders. Pediatric dAVFs typically involve the torcular herophili, superior sagittal sinuses, or large venous lakes [4]. dAVFs without aggressive angiographic features typically have a benign clinical course with a low risk of serious, lasting consequences [3-5]. dAVFs that drain from the involved dural sinus directly into the leptomeningeal veins or retrogradely into the cortical veins are associated with intracranial hemorrhage and non-hemorrhagic neurological deficits [6].

## Case presentation

**Figure 1:**
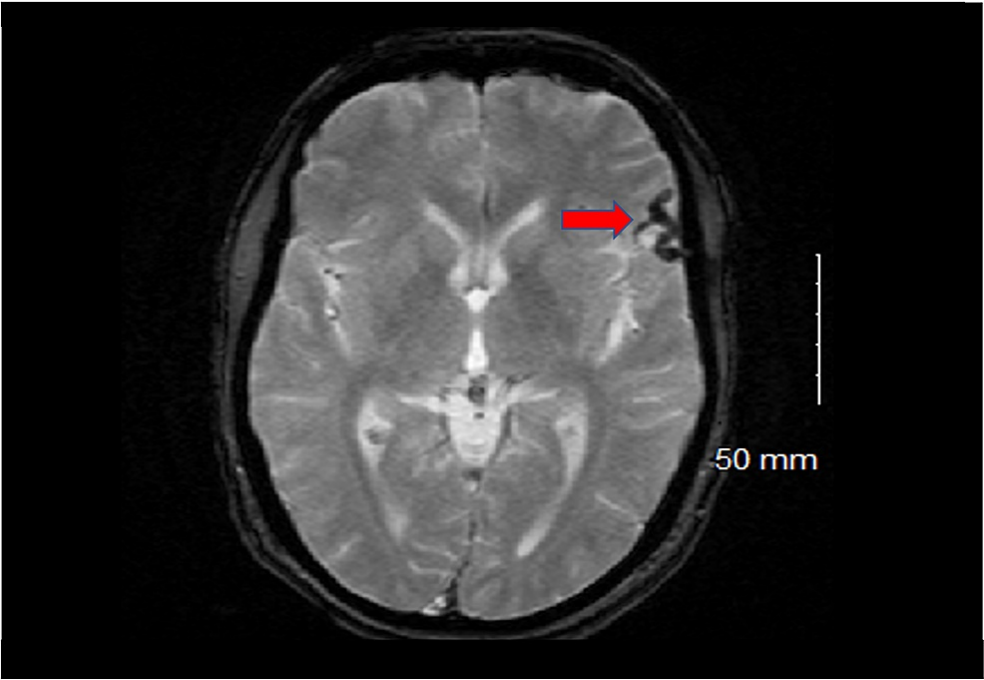
MRI of the brain before surgery A dilated Sylvian vein is visualized adjacent to the cortex of the left frontal lobe (red arrow).

A 64-year-old male with a past medical history of hypertension, hyperlipidemia, and gastroesophageal reflux disease with no past surgical history presented after an incidentally discovered Borden type III dAVF. One month before presentation to the hospital, the patient underwent outpatient brain imaging after complaining of persistent headaches. MRI of the brain revealed dilatation of the superficial middle cerebral (Sylvian) vein adjacent to the left frontal lobe, suggesting a dAVF (Figure 1).

**Figure 2:**
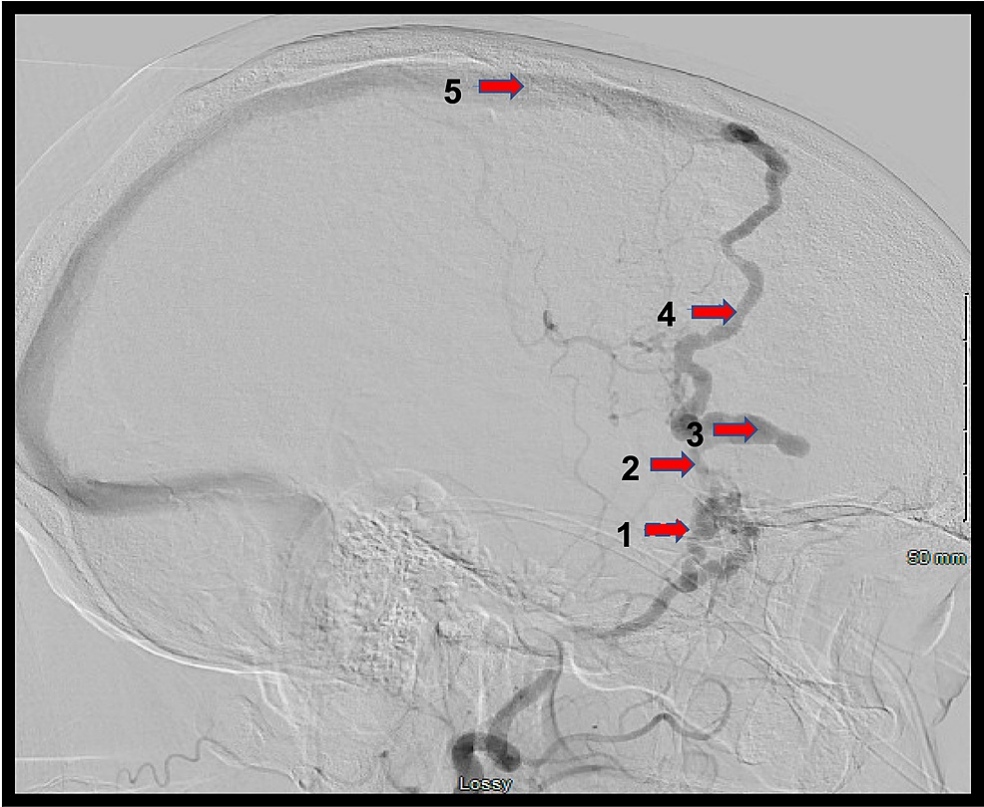
Lateral digital subtraction angiogram (DSA) of the left external carotid artery (ECA) 1: middle meningeal artery; 2: fistulous point; 3: venous ectasia; 4: Sylvian vein; 5: superior sagittal sinus

**Figure 3:**
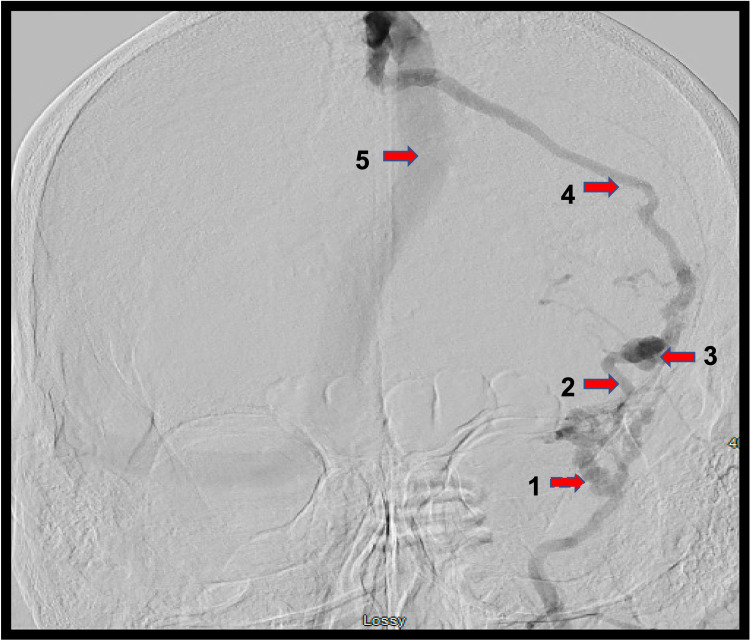
Anterior-posterior digital subtraction angiogram (DSA) of the left external carotid artery (ECA) 1: middle meningeal artery; 2: fistulous point; 3: venous ectasia; 4: Sylvian vein; 5: superior sagittal sinus

**Figure 4:**
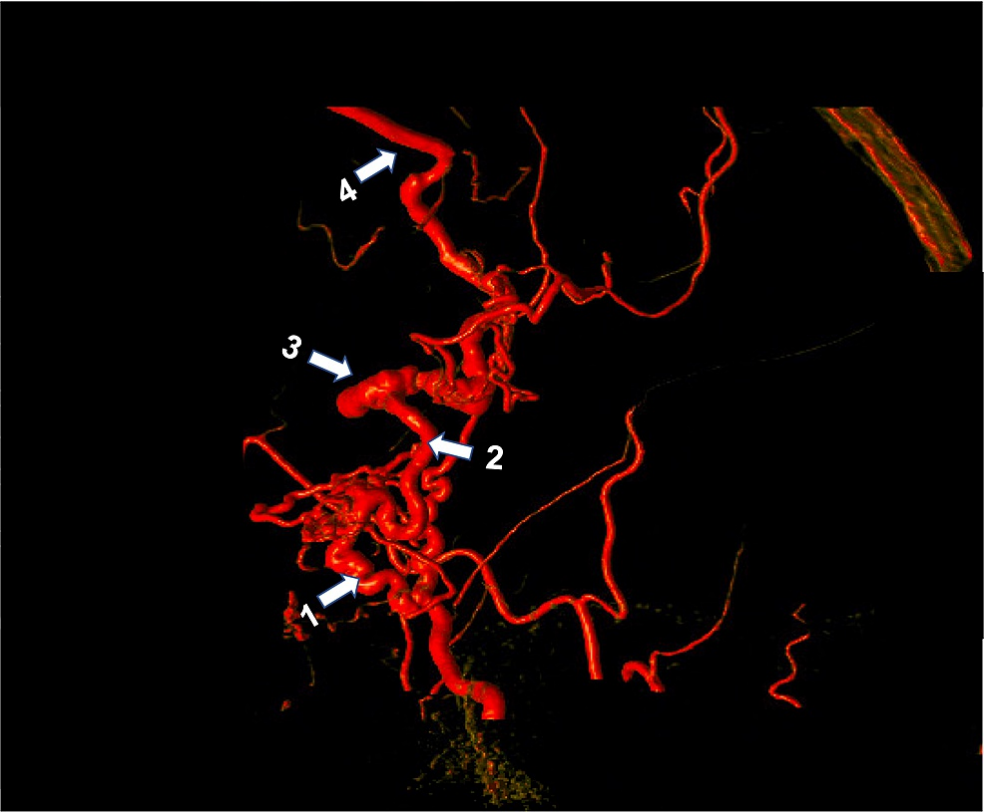
Three-dimensional rotational subtraction angiogram 1: middle meningeal artery; 2: fistulous point; 3: venous ectasia; 4: Sylvian vein

For this reason, the decision to undergo catheter angiography was made, which confirmed a dAVF arising from the anterior division of the middle meningeal artery and draining into the Sylvian vein before eventually funneling into the superior sagittal sinus. This vascular lesion corresponds to a Borden type III (Cognard type IV) dAVF (Figures 2-4).

Due to the close proximity of the fistula to the orbit and the concern for dangerous extracranial-to-intracranial anastomoses, endovascular repair of the fistula was deemed unsafe, and the patient was recommended for a craniotomy to ligate the dAVF. 

A tailored pterional craniotomy under navigation was performed and the dura was opened, revealing an enlarged, arterialized Sylvian vein. This vein followed the Sylvian fissure to the sphenoid ridge, where it entered the dura surrounded by a tangle of middle meningeal artery branches (Figures 2-3). A 10 mm permanent curved aneurysm clip was placed across the draining vein. Within a few minutes, the arterialized Sylvian vein had lost its turgor and turned blue. Complete ligation of the fistula was confirmed with indocyanine green angiography, which confirmed the absence of flow beyond the clip and no arterialized flow into the previously identified draining vein. The engorged dural branches were coagulated using bipolar electrocautery, and the vein was coagulated and cut with micro scissors distal to the clip. 

**Figure 5:**
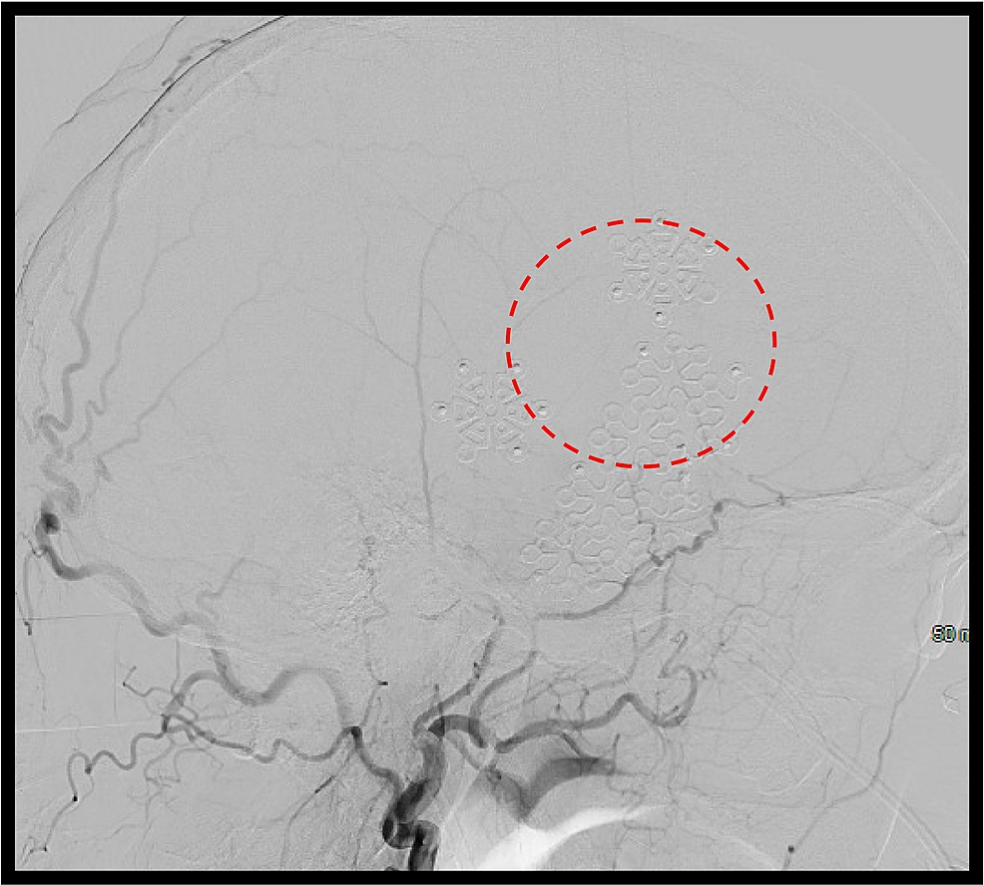
Lateral digital subtraction angiogram (DSA) of the left external carotid artery (ECA) after surgery No filling of the previously visualized fistula (red circle)

**Figure 6:**
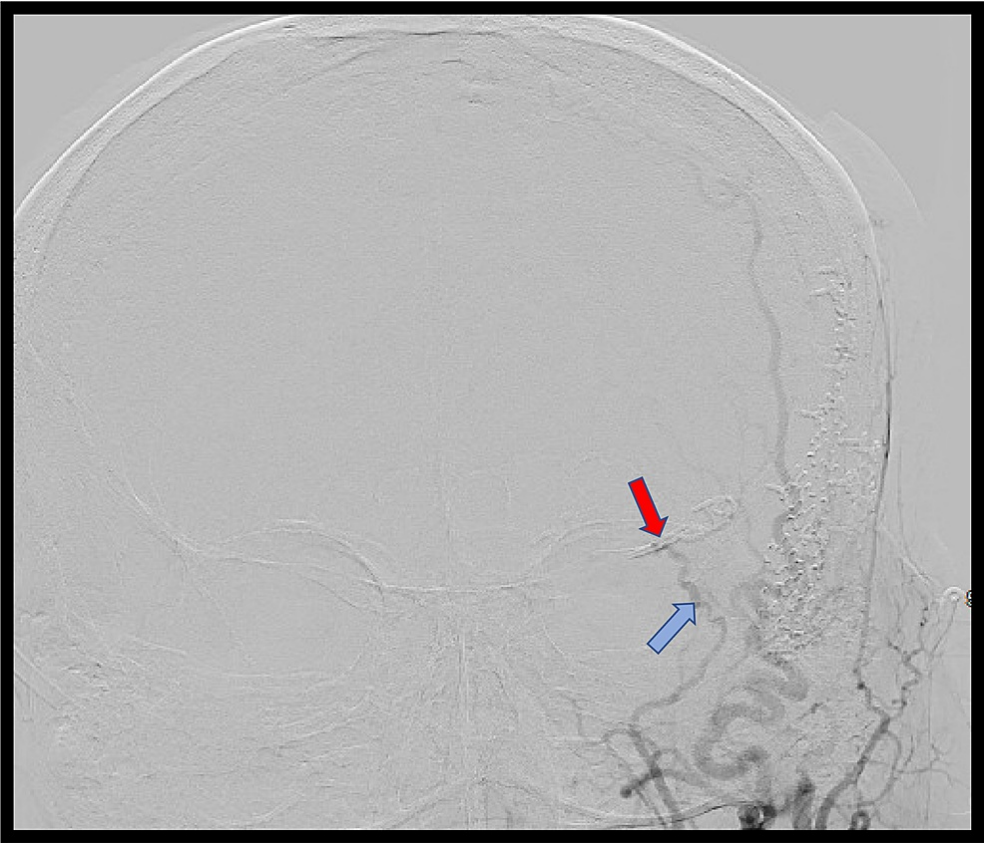
Anterior-posterior digital subtraction angiogram (DSA) of the left external carotid artery (ECA) after surgery No filling was observed beyond the clip (red arrow). The middle meningeal artery has returned to its normal caliber (blue arrow).

The postoperative period proceeded without complications, and the patient was discharged home the next day. One month later, the patient returned for follow-up. A catheter angiogram of the brain confirmed the absence of flow through the dAVF and a middle meningeal artery that had returned to its normal diameter (Figures 5-6).

## Discussion

DAVFs are dynamic lesions and fistulas which may initially lack aggressive angiographic features but can occasionally morph into aggressive lesions draining into cortical veins. Such transformations are usually secondary to venous stenosis, venous thrombosis, or increased arterial supply and are often associated with worsening clinical symptoms [7]. Reported rates of hemorrhage, non-hemorrhagic neurological deficit, and mortality associated with dAVFs exhibiting drainage via cortical veins have varied, possibly due to the lack of published research describing such cases. Shin et al. showed that most patients with benign intracranial dAVFs experience a mild disease course but in rare instances may develop cortical venous drainage secondary to progressive venous outlet obstruction [7]. This study also found that annual rates of hemorrhage, non-hemorrhagic neurological deficit, and mortality in patients with dAVFs exhibiting cortical venous drainage to be 4.5%, 7.2%, and 11.6%, respectively. Meanwhile, Borden et al. found annual rates of hemorrhage, non-hemorrhagic neurological deficit, and mortality in patients with dAVFs exhibiting cortical venous drainage to be 19.2%, 10.9%, and 19.3% respectively [8]. There are many classifications for dAVFs but the two proposed by Borden and Cognard are the most widely used [1]. The Borden classification categorizes dAVFs based on the site of venous drainage and the absence or the presence of cortical venous drainage (Table 1) [8]. 

**Table 1:** Borden Classification of Dural Arteriovenous Fistulae

Borden Classification Type	Description
Type I	Venous drainage into a dural sinus; no cortical venous drainage
Type II	Venous drainage into a dural sinus with associated cortical venous drainage
Type III	Drainage into cortical veins

The Cognard system not only notes these features but also considers the direction of flow in an involved dural sinus, as well as the absence or presence of venous ectasia (Table 2) [9].

**Table 2:** Cognard Classification of Dural Arteriovenous Fistulae

Cognard Classification Type	Description
Type I	Venous drainage into dural sinus with antegrade flow
Type II a	Venous drainage into dural sinus with retrograde flow
Type II b	Venous drainage into dural sinus with antegrade flow and cortical venous drainage
Type III	Venous drainage into cortical veins
Type IV	Cortical venous drainage with associated venous ectasia
Type V	Venous drainage into spinal perimedullary veins

Signs and symptoms of dural arteriovenous fistulas

Symptoms of dAVFs depend on their location, arterial supply, degree of arteriovenous shunting, and venous drainage [1, 5]. DAVFs that do not have drainage to the cortical veins can be asymptomatic but may also present with symptoms related to increased dural sinus blood flow, such as pulsatile tinnitus. If the cavernous sinus is involved, it can present with ocular symptoms including chemosis, ophthalmoplegia, proptosis, and decreased visual acuity due to compression of the cranial nerves found within [9-11]. DAVFs draining through the cortical veins often have a more aggressive clinical presentation, characterized by the sudden onset of severe headache, seizures, and intraparenchymal, subarachnoid, and subdural hemorrhage [5, 12-13].

Diagnosis of dural arteriovenous fistulas

The diversity in the clinical presentation of dAVFs can make their diagnosis a challenge, as most symptoms are nonspecific. In patients presenting with intracranial hemorrhage or unexplained neurological deficits, the clinician must have a high level of suspicion for dAVFs. Non-contrast CT and conventional MRI typically appear normal in asymptomatic dAVFs but may also show hemorrhage, cerebral edema, venous congestion, venous aneurysms, tortuous cortical veins, parenchymal or leptomeningeal enhancement within the dAVF, and cortical venous drainage, all of which help to establish the diagnosis of dAVF [14-15]. More recent advancements in imaging, such as time-resolved computed tomography angiography (CTA) and magnetic resonance angiography (MRA), are able to track the passage of a contrast bolus through the cerebral vasculature, further differentiating the arterial and venous phases [16]. These techniques may also permit visualization of early arteriovenous shunting into a diseased dural sinus. However, despite advancements in CT and MR imaging, catheter angiography remains the definitive imaging study for the evaluation of dAVFs because of its superior spatial and temporal resolution. Catheter angiography allows for visualization of the arterial supply and venous drainage of dAVFs, as well as their cortical venous drainage. In addition, catheter angiography is the most sensitive modality for detecting venous outflow obstruction and venous aneurysms [1].

Management and treatment of dural arteriovenous fistulas

Management of dAVFs should be based on patient characteristics, symptom severity, and the presence or absence of cortical venous reflux [1]. DAVFs without high-risk features or drainage into cortical veins may be managed conservatively with a low rate of serious complications [11]. Complete obliteration of the arteriovenous fistulas may not be necessary, but any residual fistulas could potentially recruit a new arterial supply and redevelop the fistulous connection [1]. In contrast, dAVFs with severe clinical presentations or high-risk angioarchitecture features should be promptly treated with the goal of complete fistula closure. Various treatment modalities are used to manage dAVFs, including endovascular techniques, surgery, radiosurgery, or a combination of these treatments, with the endovascular approach widely considered as the first-line treatment for dAVFs [3]. The mainstay for endovascular treatment involves embolization of the fistulous connection and its venous components while preventing adverse effects that may arise from closing a blood vessel [17]. Inappropriate embolization beyond the fistulous connection could potentially cause sudden changes in the flow dynamics and affect cortical venous drainage. While endovascular techniques are often considered primary therapy for dAVFs, in locations, such as the anterior cranial and ethmoidal fossae, surgery is thought to be more successful due to the high risk of complications associated with the endovascular approach [18]. Long-term morbidity and mortality of dAVF surgery are reported to be as high as 13%, with the major complications of surgery including infection, hydrocephalus, cerebrospinal fluid leak, stroke, cranial nerve palsy, and severe blood loss [19].

## Conclusions

DAVFs are a rare type of acquired intracranial vascular malformation whose natural course is primarily determined by the route of venous drainage. Simple fistulas can be conservatively managed if associated symptoms are minimal. In contrast, aggressive lesions with cortical venous drainage carry a significant risk of neurological deterioration and should be addressed immediately. Endovascular embolization is the main treatment modality for dAVFs, although a surgical repair may also be efficacious in some cases. In certain situations, such as when dAVFs are found in locations, such as the anterior cranial and ethmoidal fossae, a surgical approach is more successful than an endovascular approach. In the presented case, the patient had a Borden type III dAVF arising from the anterior branches of the middle meningeal artery and draining into the superficial middle cerebral vein. Due to the close proximity of the dAVF to the orbit, a surgical approach was considered to be the best treatment option.
